# Combined Treatment with Omega-3 Fatty Acid and Cholecalciferol Increases 1,25-Dihydroxyvitamin D Levels by Modulating Dysregulation of Vitamin D Metabolism in 5/6 Nephrectomy Rats

**DOI:** 10.3390/nu11122903

**Published:** 2019-12-01

**Authors:** Su Mi Lee, Mi Hwa Lee, Young Ki Son, Seong Eun Kim, Won Suk An

**Affiliations:** 1 Department of Internal Medicine, Dong-A University, Busan 49201, Korea; sumilee@dau.ac.kr (S.M.L.); kidney@dau.ac.kr (Y.K.S.); sekim@dau.ac.kr (S.E.K.); 2 Department of Anatomy and Cell Biology and Mitochondria Hub Regulation Center, Dong-A University, Busan 49201, Korea; hero15p@nate.com

**Keywords:** 1α-hydroxylase (CYP27B1), 24-hydroxylase (CYP24), 1,25-dihydroxyvitamin D, 25-hydroxyvitamin D, cholecalciferol, omega-3 fatty acid

## Abstract

The protein 1α-hydroxylase (CYP27B1) was expressed in liver and omega-3 fatty acid (FA) elevated 1,25-dihydroxyvitamin D [1,25(OH)_2_D] levels in dialysis patients. The aim of this study was to determine whether omega-3 FA and cholecalciferol have effects on vitamin D metabolism related to CYP27B1 and 24-hydroxylase (CYP24) activities in the kidney and liver of 5/6 nephrectomy (Nx) rats. Male Sprague–Dawley rats were divided into the following groups: sham control, 5/6 Nx, 5/6 Nx treated with cholecalciferol, 5/6 Nx treated with omega-3 FA, and 5/6 Nx treated with cholecalciferol/omega-3 FA. CYP27B1 and CYP24 expression were measured in the liver and kidney. Further, 1,25(OH)_2_D and 25-hydroxyvitamin D [25(OH)D] levels were measured in serum. Among Nx groups, 1,25(OH)_2_D and 25(OH)D levels were lowest in the 5/6 Nx group. CYP24 expression was increased in the kidney of the 5/6 Nx rat model, which was found to be reversed by omega-3 FA or cholecalciferol/omega-3 FA supplementation. Decreased CYP27B1 expression was observed in the liver of the 5/6 Nx rats and its expression was recovered by supplementation with cholecalciferol/omega-3 FA. In conclusion, omega-3 FA and cholecalciferol may synergistically increase 1,25(OH)_2_D levels by inhibiting CYP24 expression in the kidney and liver and activating CYP27B1 expression in the liver of 5/6 Nx rats.

## 1. Introduction

Vitamin D deficiency is very frequent among chronic kidney disease (CKD) patients and has been associated with increased mortality as well as faster progression of CKD [1,2]. Decreased renal 1α-hydroxylase (CYP27B1) activity with reduced kidney function in CKD contributes to a gradual decrease in 1,25-dihydroxyvitamin D [1,25(OH)_2_D] levels [3]. In addition, increased capacity of 24-hydroxylase (CYP24) is associated with vitamin D catabolism in CKD [3]. Therefore, CYP27B1 and CYP24 in proximal tubules of kidney play an important role in vitamin D metabolism in CKD [4].

One study reported that CYP27B1 was strongly presented in monocytes that develop into Kupffer cells [5]. Recent studies reported that CYP24 is expressed in hepatocytes [6,7]. In addition, rat models of ageing and long-term diabetes mellitus showed significantly increased expression of CYP24 in hepatocytes, as well as in non-hepatocytes including Kupffer cells, hepatic stellate cells, and sinusoidal endothelial cells [7]. Extrarenal 1,25(OH)_2_D production has previously been reported in anephric patients undergoing hemodialysis [8]. A previous human study reported that 1,25(OH)_2_D levels are increased after omega-3 fatty acid (FA) supplementation in patients with scanty renal function undergoing dialysis [9]. The exact mechanism is not clear, but it is presumed that vitamin D activation is caused by omega-3 FA supplementation. It is likely that both the kidney and liver may be involved in the vitamin D activation and catabolism. 

The aim of study is to identify whether omega-3 FA and cholecalciferol affect vitamin D metabolism related to CYP27B1 and CYP24 activities in the kidney and liver tissue of a 5/6 nephrectomy (Nx) rat model.

## 2. Materials and Methods

### 2.1. Animals and Experimental Design

A study was carried out on nine-week-old male Sprague–Dawley rats (320–350 g). The 5/6 Nx was performed on seven-week-old rats and one kidney was completely removed. One week after the first intervention, two-thirds of the remaining kidney of the animals was removed. Age-matched male Sprague–Dawley rats (350–390 g) underwent a sham procedure and they were used as control. All rats including 5/6 Nx rats were purchased from Japan SLC, Inc. (Shizuoka, Japan). The rats were housed in temperature- and light-controlled conditions and allowed free access to food and water. The protocols were approved by the Institutional Animal Care Committee of Dong-A University (DIACUC-14-4).

Rats were randomly divided into five groups of six rats each. Group 1 contained sham control rats administered saline (1 mL/kg/day) by gastric gavage for 45 days. Group 2 contained 5/6 Nx rats administered saline (1 mL/kg/day) by gastric gavage for 45 days. Group 3 contained 5/6 Nx rats administered vitamin D (cholecalciferol 3000 IU/kg/week; Solgar, Leonia, NJ, USA) by gastric gavage for 45 days. Group 4 contained 5/6 Nx rats administered omega-3 FA (Omacor, 300 mg/kg/day; Pronova Biocare, Sandefjord, Norway) by gastric gavage for 45 days. Omacor consisted of eicosapentaenoic acid 460 mg and docosahexaenoic acid 380 mg. The dose and administration route of cholecalciferol and omega-3 FA were previously described [10]. Group 5 contained 5/6 Nx rats administered cholecalciferol and omega-3 FA for 45 days. Rats were fed equally (Supplementary Figure S1), and body weight was monitored daily. At 15.4 weeks of age, the rats were anesthetized with diethyl ether anesthesia, and blood samples were obtained from the heart.

The levels of blood urea nitrogen (BUN), serum creatinine (sCr), calcium, and phosphorus were measured by an automatic analyzer (Roche, Germany). Radioimmunoassay kits (DiaSorin Inc. Stillwater, MN, USA) were used to check serum 25-hydroxyvitamin D [25(OH)D] and 1,25(OH)_2_D levels.

### 2.2. Histopathological Evaluation

Kidney tissues were fixed in 10% buffered formalin and, after dewaxing and rehydrating, sections were stained with periodic acid-Schiff. The presence of tubular atrophy, interstitial fibrosis, and inflammatory cell infiltration in kidney was assessed. All sections were imaged using Aperio ScanScope (Aperio Technologies, Vista, CA, USA). The tubulointerstitial fibrosis was quantified by using computer-assisted quantitative analysis (CaseViewer 2.2, 3DHISTECH Ltd., Budapest, Hungary).

### 2.3. Immunohistochemistry

Immunohistochemical (IHC) staining for CYP27B1 and CYP24 was carried out on a 4-μm-thick section of kidney. Kidney sections were performed using a BenchMark XT automated immunostainer (Ventana Medical System Inc., Tuscon, AZ, USA). For antigen retrieval, Cell Conditioning Solution CC1 standard buffer (pH 8.4), containing Tris/Borate/EDTA (Ventana Medical Systems) was used for 24 min at 95 °C. The tissue sections were incubated with 3% H_2_O_2_ (Ventana Medical Systems) for 4 min at 37 °C to block endogenous peroxidase activity. Next, the slides were incubated with anti-CYP27B1 (1:200; Abcam, Cambridge, UK) and anti-CYP24 (1:100; Abcam, Cambridge, UK) antibody for 32 min at 37 °C, and with the secondary antibody for 16 min at 37 °C. The slides were incubated in DAB Detection Kit (Ventana Medical Systems) and hematoxylin. Negative controls were stained under identical conditions but with buffer solution instead of the primary antibody. All sections were imaged using Aperio ScanScope. The CYP24 and CYP27B1 expression was quantified by using a computer-assisted quantitative analysis (CaseViewer 2.2, 3DHISTECH Ltd., Budapest, Hungary). In each specimen, five randomly selected non-overlapping high-power fields were acquired by using ×20-objective lens and then analyzed by using the Image-Pro Plus software. The CYP24 and CYP27B1 expression were calculated by dividing with the total stained areas in each slide.

### 2.4. Western Blot Analysis

The kidney and liver tissues were homogenized in PRO-PREP^TM^ protein extraction solution (Intron biotechnology, Seongnam, South Korea) and incubated at 4 °C for 30 min. The supernatant was collected after centrifugation for 20 min at 14,000 rpm, 4 °C. Protein concentration was assessed by a Bradford protein assay kit (Bio-Rad, Hercules, CA, USA). Twenty-five micrograms of protein was electrophoresed on 7.5–15% SDS/PAGE gels. The gels were transferred to a nitrocellulose membrane (Amersham Pharmacia Biotech, Piscataway, NJ, USA) and incubated in 1% skim milk blocking buffer overnight at 4 °C. The antibodies used included anti-CYP27B1, anti-CYP24, anti-transforming growth factor beta-1 (TGFβ-1), and anti-glyceraldehyde-3-phosphate dehydrogenase (GAPDH) from Santa Cruz Biotechnology, (Santa Cruz, CA, USA). Antibodies against β-actin and alpha smooth muscle actin (αSMA) were obtained from Sigma-Aldrich (St. Louis, MO, USA). Appropriate horseradish peroxidase-conjugated secondary antibody was incubated for 60 min at RT. Positive immunostaining was detected with the Super Signal West Pico enhanced chemiluminescence substrate (Thermo Fisher Scientific, Hudson, NH, USA). Images were digitally acquired by LAS-3000 Plus instrument (Fuji Film, Tokyo, Japan). The reactions were quantified and normalized to the β-actin control band using ImageJ 1.48q (National Institutes of Health, Bethesda MD, USA).

### 2.5. Statistical Analysis

Statistical analyses were performed using SPSS version 18.0 software (IMB Corp., Armonk, NY, USA). Data analysis was performed by using the Mann–Whitney *u* test for continuous variables, Chi-squared test for categorical variables, and Kruskal–Wallis test for continuous variables among all 5 groups. Values of *p* < 0.05 were considered statistically significant. All values are expressed as the mean ± SD.

## 3. Results

### 3.1. Characteristics of Animals

As shown in Table 1, 5/6 Nx rats exhibited significantly lower body weights than the sham control rats at the end of the experiment. In the 5/6 Nx group, levels of BUN and sCr were the highest and 25(OH)D and 1,25(OH)_2_D levels were the lowest among the five groups. Compared with levels in the 5/6 Nx group, the 25(OH)D levels were increased in 5/6 Nx rats treated with omega-3 FA. Among 5/6 Nx groups, the serum 25(OH)D and 1,25(OH)_2_D levels were the highest in 5/6 Nx rats treated with cholecalciferol and omega-3 FA. 

### 3.2. CYP24 Expression in the Kidney and Liver

**Figure 1:**
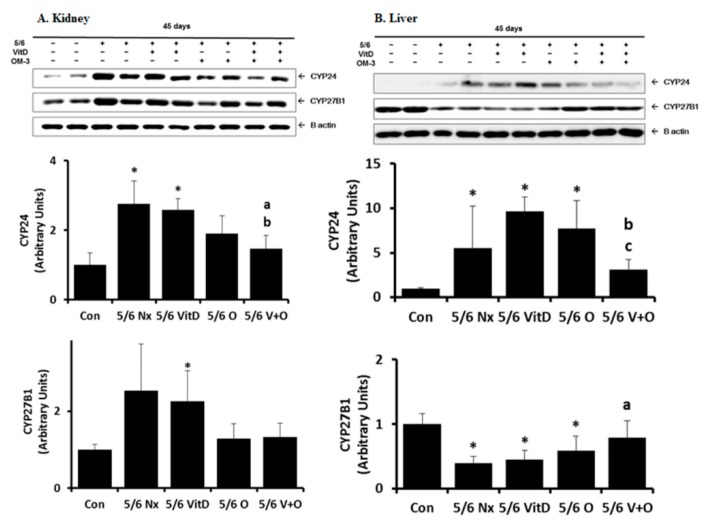
CYP24 and CYP27B1 expression in the kidney (**A**) and liver (**B**) in 5/6 nephrectomy (Nx) rats. * *p* value < 0.05, compared to the control group. ^a^ *p* value < 0.05, compared to the 5/6 Nx group. ^b^ *p* value < 0.05, compared to the 5/6 Nx with cholecalciferol group. ^c^ *p* value < 0.05, compared to the 5/6 Nx with omega-3 fatty acid group.

Figure 1 shows the results of CYP24 expression in the kidney and liver. Compared with the control group, the 5/6 Nx group showed significantly increased CYP24 expression in the kidney and liver tissue (*p* = 0.021, *p* = 0.019, respectively). Following supplementation with cholecalciferol with omega-3 FA, CYP24 expression was significantly lower than levels upon cholecalciferol monotherapy in the kidney and liver or omega-3 FA monotherapy in liver. Omega-3 FA monotherapy partially decreased CYP24 expression in the kidney. 

**Figure 2:**
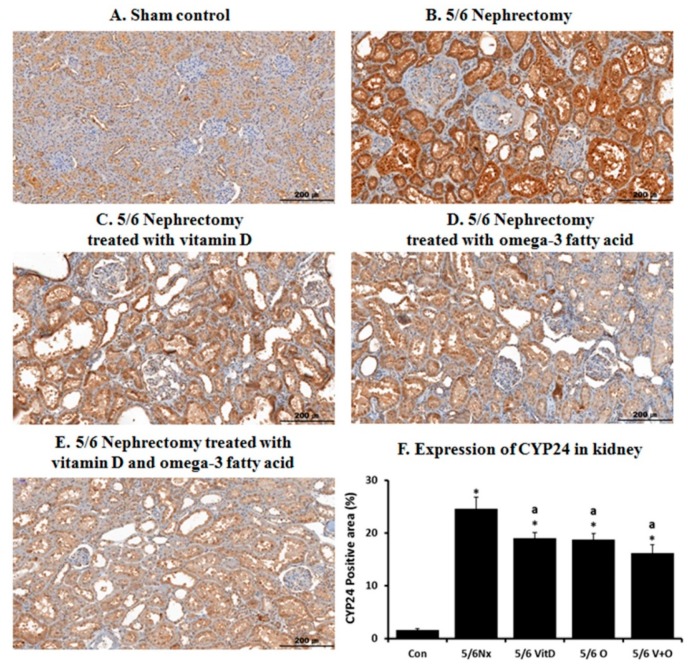
Immunohistochemical staining of CYP24 in the kidney of sham control rats (**A**), 5/6 nephrectomy (Nx) rats (**B**), 5/6 Nx rats treated with vitamin D (**C**), 5/6 Nx rats treated with omega-3 fatty acid (FA) (**D**), 5/6 Nx rats treated with cholecalciferol and omega-3 FA (**E**), and quantitative CYP24 expression differences between the groups (**F**). * *p* value < 0.05, compared to the control group. ^a^ *p* value < 0.05, compared to the 5/6 Nx rats.

Figure 2 shows the IHC staining of CYP24 expression in the kidney. CYP24 was mainly expressed in the tubules of the sham controls. Its expression level was markedly increased in the kidney of the 5/6 Nx rats. However, it was decreased upon cholecalciferol and omega-3 FA supplementation.

### 3.3. CYP27B1 Expression in the Kidney and Liver

As demonstrated by western blotting analysis, CYP27B1 levels were increased in the remnant kidney of the 5/6 Nx group when compared to levels in the control group (*p* = 0.004), the effects of which were reversed by omega-3 FA monotherapy or cholecalciferol and omega-3 FA (Figure 1). The western blots of the liver samples from the 5/6 Nx group showed a significant decrease in CYP27B1 levels when compared to the levels in the control group (*p* = 0.014). Single supplementation with cholecalciferol or omega-3 FA is little effect on CYP27B1 expression in the liver. However, cholecalciferol and omega-3 FA supplementation reinforced this down-regulation in the liver. 

**Figure 3:**
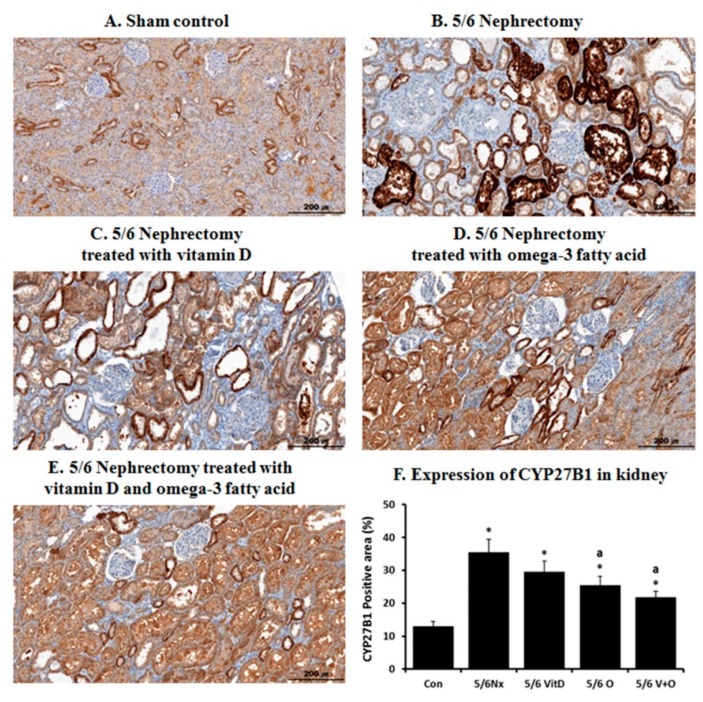
Immunohistochemical staining of CYP27B1 in the kidney of sham control rats (**A**), 5/6 nephrectomy (Nx) rats (**B**), 5/6 Nx rats treated with vitamin D (**C**), 5/6 Nx rats treated with omega-3 fatty acid (FA) (**D**), 5/6 Nx rats treated with cholecalciferol and omega-3 FA (**E**) and quantitative CYP27B1 expression differences between the groups (**F**). * *p* value < 0.05, compared to the control group. ^a^ *p* value < 0.05, compared to the 5/6 Nx rats.

Figure 3 shows the IHC staining of CYP27B1 expression in the kidney. CYP27B1 was markedly expressed in the tubules of the sham control group. Its expression level was increased in the kidney of 5/6 Nx rats, which was reversed by omega-3 FA monotherapy or cholecalciferol and omega-3 FA supplementation.

### 3.4. Renal Pathology

**Figure 4:**
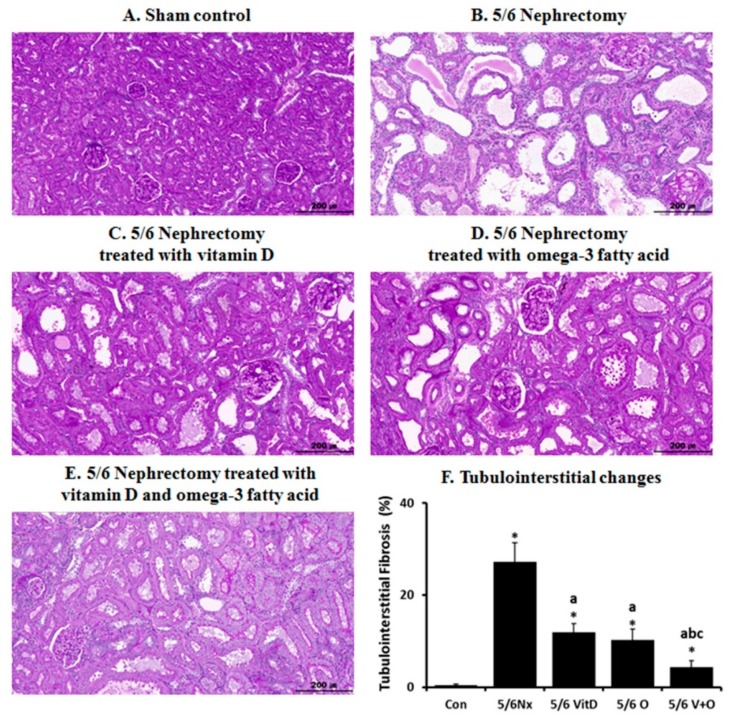
Histopathologic changes in the kidney of 5/6 nephrectomy (Nx) rats. Compared to sham control rats (**A**), 5/6 Nx rats (**B**) showed tubular dilatation, tubular atrophy, and interstitial fibrosis. In contrast, 5/6 Nx rats treated with cholecalciferol (**C**), 5/6 Nx rats treated with omega-3 fatty acid (FA) (**D**), and 5/6 Nx rats treated with cholecalciferol and omega-3 FA (**E**) showed less tubulointerstitial changes than the 5/6 Nx rats. (magnification, ×200). Quantitative tubulointerstitial changes between the groups (**F**). * *p* value < 0.05, compared to the control group. ^a^ *p* value < 0.05, compared to the 5/6 Nx group. ^b^ *p* value < 0.05, compared to the 5/6 Nx with cholecalciferol group. ^c^ *p* value < 0.05, compared to the 5/6 Nx with omega-3 FA group.

Figure 4 shows the histopathologic changes in the kidney of five experimental groups. Compared to the sham control, the 5/6 Nx group showed severe tubulointerstitial changes such as tubular dilatation, tubular atrophy, or interstitial fibrosis. The 5/6 Nx rats treated with cholecalciferol or omega-3 FA showed less tubulointerstitial changes in the remnant kidney than the 5/6 Nx rats. The tubulointerstitial fibrosis areas in kidney were increased in the 5/6 Nx rats but were decreased by omega-3 FA or cholecalciferol supplementation (Figure 4F). To identify the effect of cholecalciferol or omega-3 FA on renal fibrosis, TGFβ-1 and αSMA expression were measured via western blot analysis (Supplementary Figure S2). The levels of TGFβ-1 and αSMA were increased in the remnant kidney of the 5/6 Nx group. However, it was decreased upon cholecalciferol and omega-3 FA supplementation.

## 4. Discussion

In this study, we found that cholecalciferol and omega-3 FA supplementation increased 1,25(OH)_2_D levels in a 5/6 Nx rat model. Interestingly, CYP24 expression was increased in both the kidney and liver tissues of the 5/6 Nx rat model, which was found to be reversed only by combined cholecalciferol and omega-3 FA supplementation. In addition, decreased CYP27B1 expression was observed in the liver of the 5/6 Nx rats and its expression was recovered by combined treatment with cholecalciferol and omega-3 FA. Notably, the increased expression of 24-hydroxylase observed not only in the remnant kidney but also in the liver may explain the decreased serum 1,25(OH)_2_D levels in CKD. Furthermore, decreased CYP27B1 expression in the liver may be involved in vitamin D activation in this CKD model. Therefore, it is necessary to consider the role of the liver in vitamin D metabolism in CKD.

CYP27B1 activity is influenced by several factors, such as 1,25(OH)_2_D, calcium, phosphorus, parathyroid hormone, and fibroblast growth factor-23 (FGF-23) levels. CYP27B1 is primarily expressed in renal proximal and distal convoluted tubules, but it is also expressed in extrarenal tissues, such as the skin, lymph nodes, colon, pancreas, adrenal medulla, brain, placenta, lymphocytes, and macrophages [11,12,13]. The role of CYP27B1 at extrarenal sites is unclear, but functions related to autocrine or paracrine vitamin D modulation may exist. Decreased renal mass limits the amount of CYP27B1 or the delivery of 25(OH)D to CYP27B1, resulting in reduced production of 1,25(OH)2D [14,15]. However, CYP27B1 levels in patients with CKD were not decreased in some cases, suggesting that another mechanism such as extrarenal activation may explain the change of vitamin D levels in CKD [3,16].

In our study, increased renal CYP27B1 expression with low vitamin D levels were observed in 5/6 Nx rats. These findings suggest that CYP27B1 may be excessively expressed to compensate for the decreased kidney mass in 5/6 Nx rats, but its ability to maintain 1,25(OH)2D levels was inadequate. On the contrary, omega-3 FA and cholecalciferol supplementation nearly normalized renal CYP27B1 expression in the remaining kidney tissue of 5/6 Nx rats. This finding can be explained by decreased catabolism of active vitamin D, resulting in adequate 1,25(OH)2D levels. Meanwhile, CYP27B1 expression in the liver tissues of 5/6 Nx rats was suppressed, and omega-3 FA and cholecalciferol supplementation nearly recovered CYP27B1 expression in the liver of 5/6 Nx rats. These results suggested that omega-3 FA supplementation partially up-regulates CYP27B1 expression, and cholecalciferol helped upregulate CYP27B1 in the liver of 5/6 Nx rats based on the effectiveness of combined supplementation.

CYP24, a member of the cytochrome P450 enzyme superfamily, catalyzes hydroxylation reactions, which initiate the degradation of 1,25(OH)_2_D to form biologically inactive water-soluble calcitroic acid. Previous studies have reported an inverse relationship between renal CYP24 expression and 1,25(OH)_2_D levels [17,18]. Elevated renal CYP24 expression levels and low vitamin D levels in 5/6 Nx rats may reflect the accelerated catabolism of 1,25(OH)_2_D and 25(OH)D by CYP24. This overexpression of CYP24 in the liver and kidney tissues of Nx rats was synergistically ameliorated by combined treatment with cholecalciferol and omega-3 FA, but not by single treatment.

In previous studies, supplementation with omega-3 FA or omega-3 FA and cholecalciferol elevated 1,25(OH)2D levels in patients undergoing dialysis [9,17]. Our data support that increased 1,25(OH)_2_D concentrations were caused by CYP24 inactivation in the kidney and liver tissues and CYP27B1 activation in the liver after cholecalciferol and omega-3 FA supplementation. Notably, omega-3 FA supplementation alone significantly increased 25(OH)D levels and slightly increased 1,25(OH)2D levels in this study. The increased 25(OH)D levels without cholecalciferol supplementation may be related to 25-hydroxylase activity. Further studies will be needed to confirm the effect of cholecalciferol and omega-3 FA on vitamin D metabolism in patients with CKD.

A limitation of this study was that IHC staining for CYP24 and CYP27B1 was not performed in the liver tissues. Therefore, we cannot confirm the expression of CYP24 and CYP27B1 in hepatocytes or non-hepatocyte liver cells. Additional studies are needed to elucidate CYP24 and CYP27B1 expression in the liver. Further experiments conducted in cell and animal models are required to unravel the intriguing contribution of omega-3 FA to vitamin D metabolism in kidney disease.

## 5. Conclusions

Cholecalciferol and omega-3 FA supplementation increased serum vitamin D levels by modulating CYP24 and CYP27B1 expression in the kidney and liver tissues of 5/6 Nx rats. Further studies are required to explicate the crosstalk between the liver and kidney with regard to CYP24 and CYP27B1 activities in this CKD rat model.

## Figures and Tables

**Table 1:** Biochemical parameters.

	Control (*n* = 6)	5/6 Nephrectomy (*n* = 6)	5/6 Nephrectomy with Cholecalciferol (*n* = 6)	5/6 Nephrectomy with Omega-3 FA (*n* = 6)	5/6 Nephrectomy with Cholecalciferol and Omega-3 FA (*n* = 6)	*p* Value
Final body weight (g)	464 ± 9	385 ± 11 *	420 ± 33 *	422 ± 10 *^a^	422 ± 12 *^a^	<0.001
Body weight gain (g)	91 ± 3	46 ± 13 *	71 ± 26	75 ± 4 ^a^	73 ± 6 ^a^	<0.001
Blood urea nitrogen (mg/dL)	18 ± 0.7	77 ± 35 *	72 ± 23 *	68 ± 19	55 ± 13 ^ab^	0.003
Creatinine (mg/dL)	0.4 ± 0.0	1.3 ± 0.6 *	1.2 ± 0.3 *	1.1 ± 0.3	0.9 ± 0.2 ^abc^	0.002
Calcium (mg/dL)	7 ± 0.4	7 ± 1	6 ± 0.4	8 ± 0.6	7 ± 1	0.50
Phosphorus (mg/dL)	8 ± 0.5	11 ± 5	8 ± 1	8 ± 0.6	8 ± 0.6	0.26
25(OH)D (ng/mL)	98 ± 6	28 ± 17 *	50 ± 42	62 ± 39 ^a^	111 ± 37 ^abc^	0.003
1,25(OH)_2_D (pg/mL)	171 ± 41	45 ± 17^*^	58 ± 41 *	83 ± 42 *	108 ± 43 *^ab^	0.002

25(OH)D, 25-hydroxyvitamin D; 1,25(OH)2D, 1,25-dihydroxyvitamin D; FA, fatty acid. Values represent the means ± SD. The nonparametric Wilcoxon exact rank sum test or Mann–Whitney U test was used. * *p* value < 0.05, compared to the control group. ^a^ *p* value < 0.05, compared to the 5/6 nephrectomy (Nx) group. ^b^ *p* value < 0.05, compared to the 5/6 Nx with cholecalciferol group. ^c^ *p* value < 0.05, compared to the 5/6 Nx with omega-3 fatty acid group.

## Supplementary Materials

The following are available online at https://www.mdpi.com/2072-6643/11/12/2903/s1, Figure S1: Amount of food intake in different groups, Figure S2: Expression of TGFβ-1 and αSMA. * *p* value < 0.05, compared to the control group. ^a^ *p* value < 0.05, compared to the 5/6 Nx group.
