# Supplementary material for: Combined Treatment with Omega-3 Fatty Acid and Cholecalciferol Increases 1,25-Dihydroxyvitamin D Levels by Modulating Dysregulation of Vitamin D Metabolism in 5/6 Nephrectomy Rats
Source: Nutrients. 2019 Dec 1;11(12):2903. doi: 10.3390/nu11122903 (PMC6950759; doi:10.3390/nu11122903)

Supplementary Figure 1. Amount of food intake in different groups

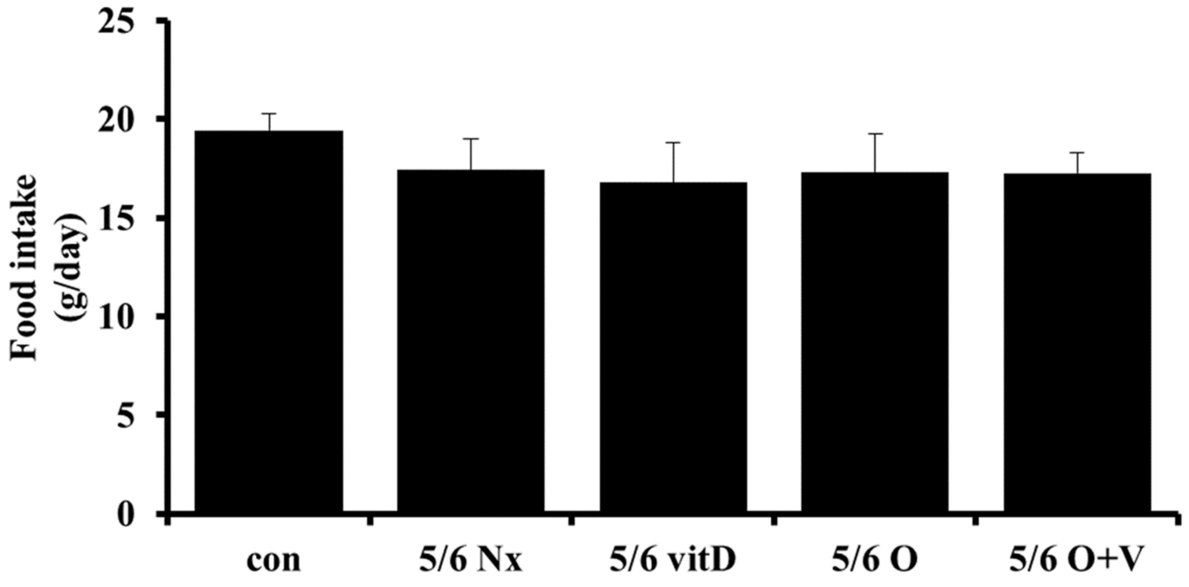

Supplementary Figure 2. Expression of TGFβ-1 and αSMA. [*]P value <0.05, compared to the control group. [a]P value <0.05, compared to the 5/6 nephrectomy group.

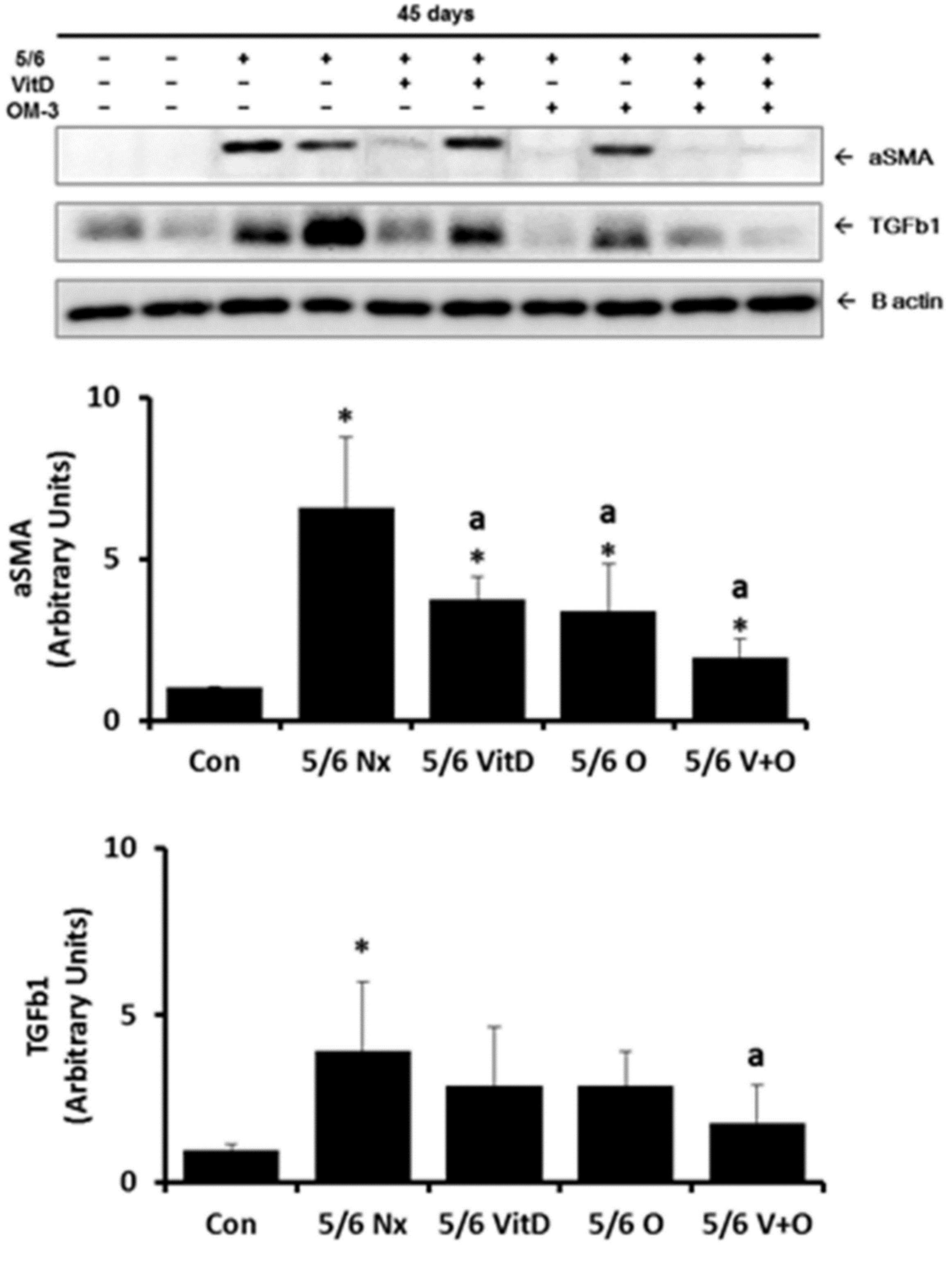

Supplement: Supplementary file 1 [file nutrients-11-02903-s001.pdf]
